# Nucleosome positions establish an extended mutation signature in melanoma

**DOI:** 10.1371/journal.pgen.1007823

**Published:** 2018-11-28

**Authors:** Alexander J. Brown, Peng Mao, Michael J. Smerdon, John J. Wyrick, Steven A. Roberts

**Affiliations:** 1 School of Molecular Biosciences, Washington State University, Pullman, WA, United States of America; 2 Center for Reproductive Biology, Washington State University, Pullman, WA, United States of America; Sun Yat-sen University, CHINA

## Abstract

Ultraviolet (UV) light-induced mutations are unevenly distributed across skin cancer genomes, but the molecular mechanisms responsible for this heterogeneity are not fully understood. Here, we assessed how nucleosome structure impacts the positions of UV-induced mutations in human melanomas. Analysis of mutation positions from cutaneous melanomas within strongly positioned nucleosomes revealed a striking ~10 base pair (bp) oscillation in mutation density with peaks occurring at dinucleotides facing away from the histone octamer. Additionally, higher mutation density at the nucleosome dyad generated an overarching “translational curvature” across the 147 bp of DNA that constitutes the nucleosome core particle. This periodicity and curvature cannot be explained by sequence biases in nucleosomal DNA. Instead, our genome-wide map of UV-induced cyclobutane pyrimidine dimers (CPDs) indicates that CPD formation is elevated at outward facing dinucleotides, mirroring the oscillation of mutation density within nucleosome-bound DNA. Nucleotide excision repair (NER) activity, as measured by XR-seq, inversely correlated with the curvature of mutation density associated with the translational setting of the nucleosome. While the 10 bp periodicity of mutations is maintained across nucleosomes regardless of chromatin state, histone modifications, and transcription levels, overall mutation density and curvature across the core particle increased with lower transcription levels. Our observations suggest structural conformations of DNA promote CPD formation at specific sites within nucleosomes, and steric hindrance progressively limits lesion repair towards the nucleosome dyad. Both mechanisms create a unique extended mutation signature within strongly positioned nucleosomes across the human genome.

## Author summary

UV-induced mutations are abundant and heterogeneously distributed across melanoma genomes. Understanding the mechanisms that produce this heterogeneity may help decipher which mutations drive the cancer phenotype. While it is known that mutation density correlates with chromatin compaction on a large scale, recent studies have suggested that local chromatin structure impacts mutation distribution in ways previously undetected. We therefore examined the distribution of melanoma mutations in strongly positioned nucleosomes where we observed a striking oscillatory and curvature pattern. UV lesion formation appeared to be responsible for mutation oscillation, despite active repair occurring in the nucleosome core particle. However, more CPD lesions are removed near the edges of nucleosomes, and thus generated an overall translational curvature in mutation density.

## Introduction

UV light causes the formation of cyclobutane pyrimidine dimers (CPDs) and, to a lesser extent, 6–4 photoproducts (6-4PPs) [1], which can induce mutations that promote the development of melanomas and other skin cancers [2]. Whole genome sequencing of melanomas has revealed that most somatic mutations in these cancers match a UV mutational signature, consisting of C -> T substitutions occurring in lesion-forming dipyrimidine sequences [3, 4]. Due to UV-induced mutagenesis, cutaneous melanomas typically have an extremely high number of base substitutions [5]. These somatic mutations are unevenly distributed across the cancer genome [6–10], despite little to no selective pressure occurring on the vast majority of these genetic changes. The high frequency and heterogeneous distribution of somatic mutations in cutaneous melanomas confound the ability to accurately identify “driver” mutations based on local abundance and recurrence, especially for less common driver mutations [2, 7]. Hence, to better understand the molecular etiology of human skin cancers, it is important to elucidate the mechanisms that shape the genomic “landscape” of UV-induced mutation.

Chromatin structure is also variable across the genome, regulating cellular processes like transcription, DNA repair, and replication in a cell-type specific manner. Effects of chromatin on mutagenesis have been observed on the global scale, where regions of compact chromatin correlate with elevated mutation density [7, 9], and on the local scale, where transcription factor (TF) binding [11–15] and individual nucleosomes [16] are associated with variations in mutation density. The impact of chromatin organization on mutation heterogeneity has largely been attributed to inhibition of DNA repair processes by occluding access to DNA lesions [17, 18]. This assessment has assumed that lesion formation is homogeneous across the genome. However, lesion formation can vary within defined structures of chromatin, such as TF binding sites [15, 19, 20], suggesting that DNA repair efficiency may not be the sole factor affecting mutation rates.

**Fig 1:**
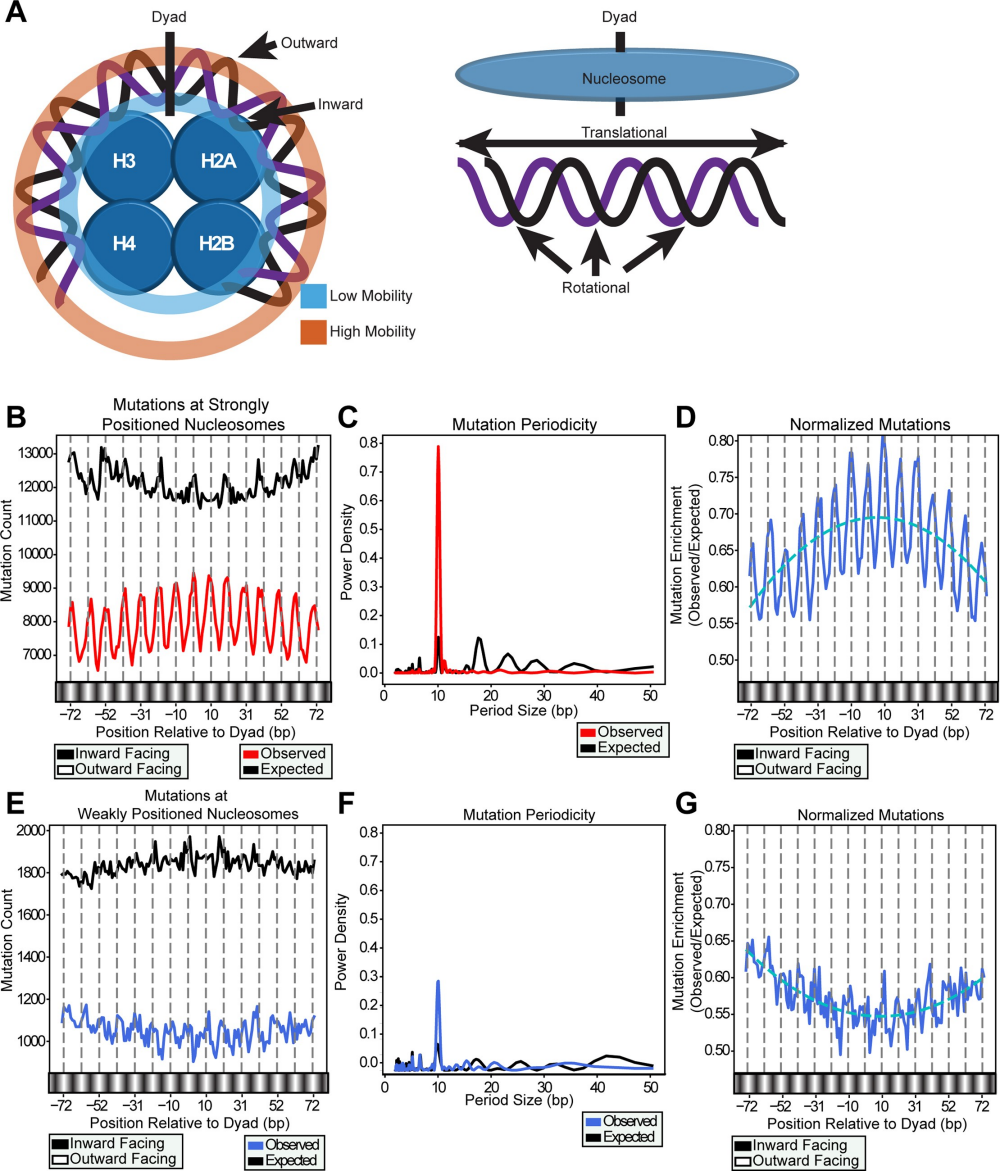
Mutations counted at strong nucleosome core particle DNA positions. (*A*) DNA wraps around the nucleosome histones (shown as blue circles), with the least accessible region near the dyad and the most accessible regions near the edges of the nucleosome (nucleosomes consist of 8 histones and almost 2 complete DNA wraps, but only 4 histones and most of 1 wrap is shown here for simplicity). The linear positioning of the DNA along the nucleosome is the translational setting. As the DNA rotates around the nucleosome, bases proximal to the histones are termed “inward” facing, while those that are distal are called “outward” facing. The inward bases experience less mobility due to the increased interactions with the histones, whereas the outward bases have greater mobility. Hence, the DNA’s rotational setting causes some bases to have higher and lower mobility. *(B)* Observed single nucleotide substitutions and expected mutations (solid lines) based on sequence context were counted at individual base pairs across nucleosome positions. Grey dashed lines indicate the outward rotational setting of the DNA, occurring every 10.3 bp. (*C*) The periods in the observed and expected mutations were quantified by Lomb-Scargle analysis. (*D*) Observed mutations normalized to the expected mutations (i.e. mutation enrichment) displays an emphasized ~10 bp periodicity as well as a “negative” curvature across the nucleosome. We represent this curvature mathematically by fitting the enrichment data to a second order best-fit polynomial [by the formula y = ax^2^ + bx + c] (dashed blue line). (*E*) Neither observed nor expected mutations at weakly positioned nucleosomes showed an obvious pattern. (*F*) The periodogram shows a slight peak at ~10 bp, which is less than half as strong as the peak observed in strongly positioned nucleosomes (Fig 1C). (*G*) The enrichment of observed to expected mutations at weakly positioned nucleosomes also does not show a significant pattern, and the curvature is inversed.

Nucleosomes are the fundamental unit of chromatin [21, 22], but the potential impact of nucleosome structure on mutation rates in melanoma is not well understood. It has been shown that in the flanking DNA around transcription factor binding sites (TFBS) nucleosomes may generate a phasing pattern in mutation density in melanoma [18]. Moreover, *in vitro* and *in vivo* studies indicate that histone-DNA contacts within individual nucleosomes modulate the formation of UV-induced CPD lesions across the 147 bp of DNA that is bound by the nucleosome core particle [19, 23, 24]. CPD formation peaks every ~10.3 bp within nucleosomal DNA, indicating that the rotational setting of DNA along the nucleosome can affect lesion formation (Fig 1A) [16]. However, it is not clear to what extent nucleosome positioning in the human genome affects CPD formation, nor if this mechanism affects mutation rates in human skin cancers. Lesion removal by the nucleotide excision repair (NER) pathway in the yeast *Saccharomyces cerevisiae* also occurs more slowly towards the center of the nucleosomes where DNA is strongly bound, and more efficiently at the edge of the nucleosome where DNA is flexible [25–27]. This indicates the linear position (translational setting) of the DNA along the nucleosome may also play a role in dictating mutation distribution.

To investigate whether individual nucleosomes modulate mutation density in human cancers, we analyzed the positions of melanoma mutations within strongly positioned nucleosomes across the human genome [28]. We show that mutation density in melanoma has a unique oscillatory pattern in strongly positioned nucleosomes, with peaks in mutation density occurring at regular ~10 bp intervals at outward rotational settings in nucleosomes. The relative contributions of lesion formation and repair in generating this pattern were assessed and revealed that lesion formation is likely responsible for the ~10 bp periodicity, while nucleotide excision repair (NER) activity appears to generate an overall “translational curvature” in mutation density across the nucleosome (i.e. higher mutation density near the dyad of nucleosomes than at the edges). We additionally parsed nucleosomes by chromatin state [29], histone modification (Roadmap Epigenomics), and transcription levels [30]. We note the periodicity in mutation density was maintained across nucleosomes regardless of these additional factors. However, nucleosomes within different chromatin states or containing pre-existing histone modifications associated with active transcription displayed differences in mutation translational curvature, revealing the time nucleosomes spend occupying DNA further dictates mutation density.

## Results

### Strongly positioned nucleosomes exhibit rotational and translational effects on mutation density

To determine the impact of nucleosome structure on mutation heterogeneity, we profiled the positions of ~21 million mutations across individual DNA base pairs within the 147 bp “core particle” that surround 1.4 million strong nucleosome dyad positions obtained from a nucleosome map derived from DNase-seq data [28]. DNase I digestion has long been used to map nucleosome DNA (e.g., [31–33]), and is particularly useful for mapping the rotational settings of nucleosomes. In contrast, MNase digestion (and MNase-seq data) is generally less accurate in defining the rotational settings of nucleosomes (e.g., see [16]). From this map, we restricted our analysis to nucleosomes displaying high positioning scores. A score of 10 or greater was chosen empirically as a threshold for strongly positioned nucleosomes, reflecting ≥10-fold higher likelihood that there is a positioned nucleosome at that location relative to the nucleosome-free background. Melanoma mutations within strongly positioned nucleosomes showed a pronounced ~10 bp periodicity (determined by Lomb-Scargle analysis) (Fig 1B and 1C) with peaks corresponding to outward facing nucleotides and dips corresponding to inward positions. Additionally, there was a slight curvature across the nucleosomal DNA, with more mutations near the central dyad. To assess whether the observed mutation pattern could be accounted for by sequence context, we calculated the expected per-nucleotide mutation density based on the trinucleotide contexts of all mutations (see Materials and Methods). In contrast to the pattern of observed mutations, the overall expected mutation distribution was elevated, due to strongly positioned nucleosomes having a reduced mutation density compared to the rest of the genome (S1 Fig). This reduction is likely due to strongly positioned nucleosomes occurring frequently in transcribed regions of the genome which are known to have lower mutation density [34]. Moreover, the expected mutation distribution failed to produce any apparent oscillation and displayed a slightly opposing translational curvature across the entirety of the nucleosome core particle (Fig 1B and 1C). The stark difference between the observed and expected mutation distributions indicate that the 10 bp periodicity in the observed mutation density as well as the translational curvature across the nucleosome core particle are likely controlled by the presence of the histone octamer on the DNA instead of the underlying DNA sequence. In accordance with this interpretation, normalization of the observed mutation density by the expected mutation density (i.e. to remove any residual effects of sequence context; referred to hereafter as a “mutation enrichment”) revealed a strong enrichment of mutations at outward rotational settings (as expected) and a striking translational curvature in the mutation density, with peak mutation density near the nucleosome center and lower mutation densities near the edges of the nucleosome (Fig 1D). This curvature can be represented by a best-fit polynomial (i.e. y = ax^2^ + bx + c) and since the primary coefficient for the polynomial describing mutation enrichment is negative, we hereafter refer to this mutation pattern as a “negative curvature.”

Further supporting that the oscillation and curvature in mutation density across strongly positioned nucleosomes is a function of specific histone-DNA contacts, the observed mutations in weakly positioned nucleosomes (i.e. positioning scores of -5 to -40) showed a much weaker oscillatory pattern (Fig 1E–1G). This is reflected in the ~3-fold lower peak of 10 bp periodicity, compared to strongly positioned nucleosomes (Fig 1F). This indicates that weakly positioned nucleosomes do not impact mutation distributions as dramatically as strongly positioned nucleosomes. After normalizing the observed mutations to those expected, the mutation enrichment across weakly positioned nucleosomes was decreased near the nucleosome center (Fig 1G), which is opposite of the pattern observed for strongly positioned nucleosomes. These results suggest that strongly positioned individual nucleosomes are associated with a unique mutation signature, with peaks in mutation density at outward rotational settings in the nucleosomal DNA, and an enrichment in mutation density near the central nucleosome dyad axis (Fig 1).

### Mutational effects at nucleosomes are driven by UV-Light

**Fig 2:**
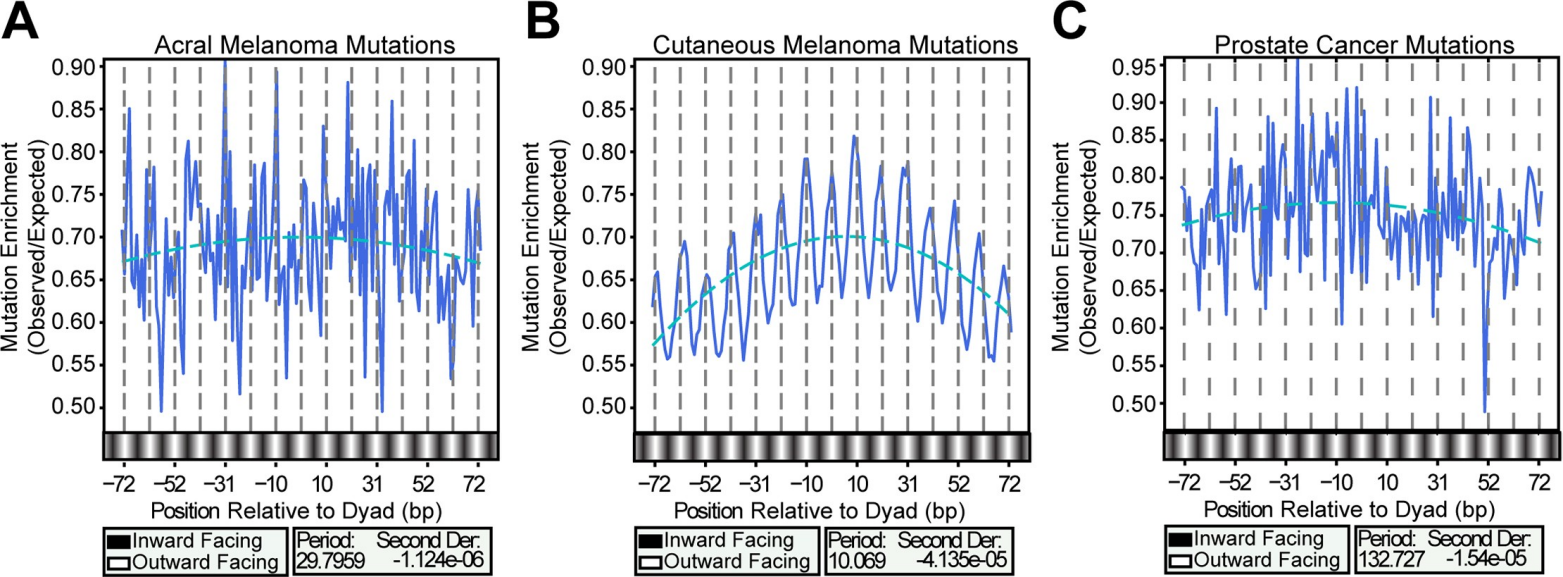
Effect of melanoma subtype on nucleosome-associated mutation patterns. Enrichments of mutations occurring in dipyrimidines in (*A*) acral melanomas (*B*) cutaneous melanomas, or (*C*) prostate cancer were calculated at nucleosome positions. The cutaneous, but not acral or prostate, mutation enrichment exhibits the same ~10 bp periodicity. As shown in S2 Fig, this was not due to a difference in power.

The main mutagenic process in melanoma derives from UV-induced DNA lesions [2]. To test the hypothesis that the mutational patterns observed in nucleosomes are caused by a mechanism involving UV lesions, we parsed the mutations occurring in dipyrimidine sequences into cutaneous (UV exposed) and acral (typically not UV exposed) melanoma subtypes [3]. We repeated the analyses evaluating mutation distributions within strongly positioned nucleosomes for each tumor subset. Mutation enrichment from acral melanoma lacked the internal 10 bp oscillation, with the most prominent periodicity at ~30 bp, and showed only a slight negative curvature across the core particle (Fig 2A). In contrast, the cutaneous mutations recapitulated the strong ~10 bp oscillation and negative translational curvature (Fig 2B), indicating that both are derived from UV damage. The acral melanomas contained ~100-fold fewer mutations than cutaneous melanomas, which might make it difficult to detect these mutational patterns in acral melanomas due to the lower total number of mutations. We therefore took 1000 random subsets of the cutaneous mutations (each subset containing ~1/100 mutations to match the number of mutations in acral tumors) to test whether the loss of periodicity in the acral tumors was potentially due to a loss of power. We calculated the periodicity for each subset and counted how many subsets exhibited the same periodicity. The vast majority of the cutaneous melanoma subsets (99.3%) had the same ~10 bp periodicity, indicating that despite the ~100-fold difference in the number of acral and cutaneous mutations, a sufficient number of mutations were present within the acral melanomas to observe any periodicity if it were to exist (S2 Fig). Similar to mutations from acral melanomas, mutations occurring in dipyrimidine sequences from non-UV-exposed prostate cancers failed to produce any significant oscillation (Fig 2C). We conclude that the oscillatory pattern of mutation density in nucleosomes is a unique feature of the UV-induced mutagenesis of cutaneous melanomas.

### CPD formation and NER activity respectively establish rotational oscillation and translational curvature in mutation density at nucleosomes

**Fig 3:**
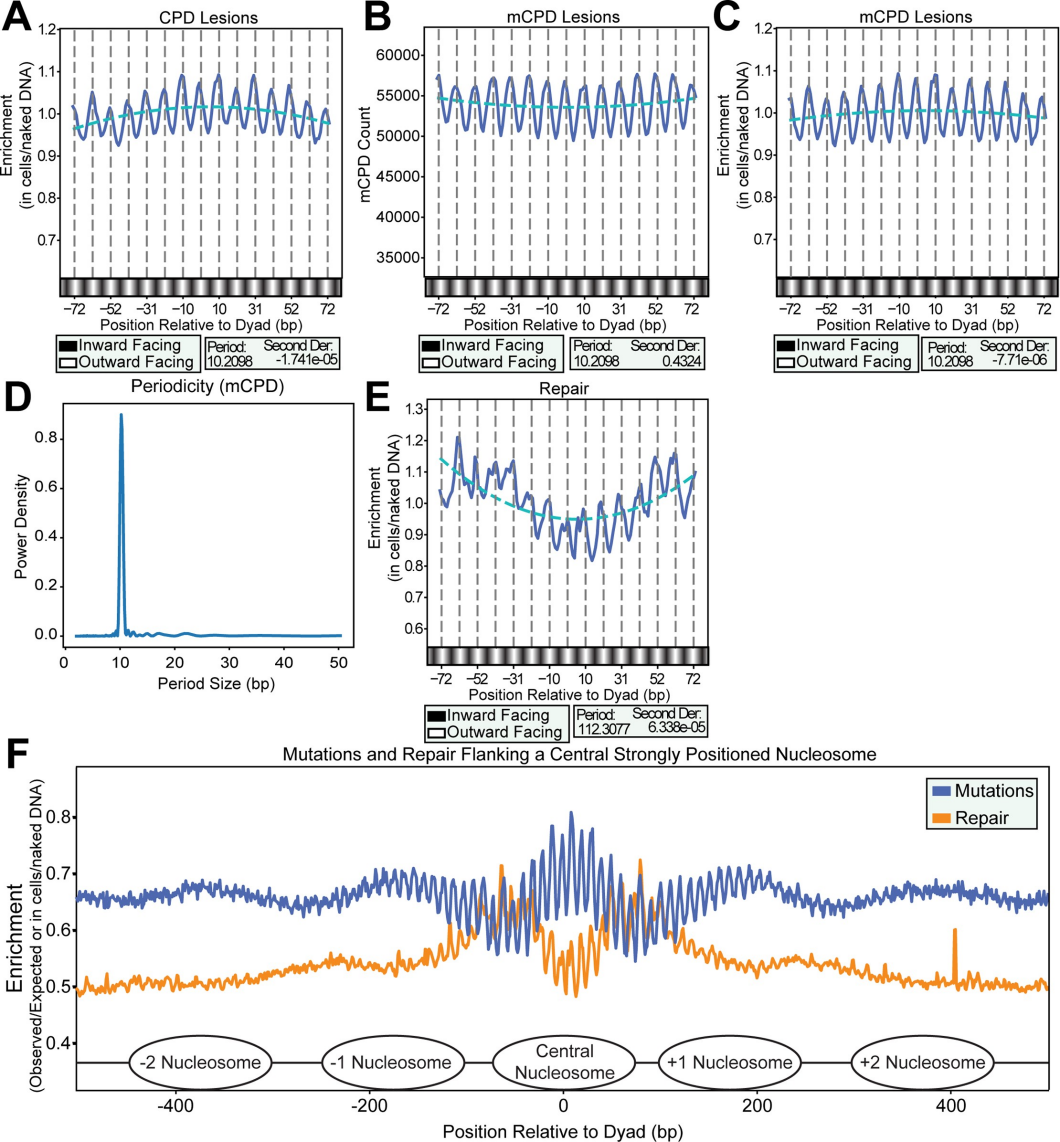
CPD lesions and repaired CPD lesions counted at nucleosome positions. (*A*) The “in cells” CPD lesion counts normalized to *in vitro* lesion counts (CPD-seq by CPD-seq; also normalized for read counts) at nucleosome positions showed the same periodicity observed in the mutation data. They also showed a slight negative curvature across the nucleosome. (*B*) The mCPD lesion counts and (*C*) mCPD lesions normalized to *in vitro* lesion counts showed the same periodicity as all CPDs (*D*), however, they showed an even more shallow negative curvature. (*E*) The in cells CPD repair counts normalized to *in vitro* CPD lesion counts (XR-seq by HS-Damage-seq; also normalized for read counts) at nucleosome positions appeared to have the same periodicity. However, the most significant periodicity by Lomb-Scargle analysis is ~112 bp. The repair counts also showed an opposing opposite curvature. (*F*) Normalized melanoma mutations and XR-seq counts at 1 hr repair across a 1000 bp window centered on a central nucleosome dyad. Nucleosome positions determined by DNase-seq are depicted graphically.

The specificity of the rotational oscillation and translational curvature in mutations across nucleosomes to cutaneous melanoma raised the question as to whether these patterns were a result of variations in lesion formation, DNA repair, or both. To examine the effects of nucleosome structure on lesion formation, we analyzed the genome-wide distribution of CPD lesions (generated by CPD-seq) in human fibroblasts (NHF1 cells) irradiated with 100J/m^2^ of UVC light [15]. We determined the number of CPD lesions that occurred at each base across the 147 bp at strongly positioned nucleosomes and divided these values by similarly acquired lesions from purified genomic DNA treated directly with 80J/m^2^ of UVC light (a dose empirically determined to yield similar levels of CPDs compared to the in cell treatment). This normalization removes variation in CPD formation based on the intrinsic DNA sequence effects [21]. Each data set was also divided by their total number of reads mapping to dipyrimidines in strongly positioned nucleosomes to account for differences in sequencing depth. This analysis of CPDs within strongly positioned nucleosomes revealed the same ~10 bp rotational pattern with peaks in normalized CPD formation at outward facing dinucleotides (Fig 3A), as observed with melanoma mutations. Additionally, as most melanoma mutations are C -> T (~90%), we next specifically analyzed potentially mutagenic cytosine-containing CPDs (mCPDs; i.e. TT CPDs were removed) and observed a similar ~10 bp rotational pattern in both raw mCPD count and mCPD enrichment (Fig 3B–3D). This analysis indicates that elevated CPD (and mCPD) formation at outward rotational settings in strongly positioned nucleosomes is likely responsible for elevated mutagenesis at these same sites in cutaneous melanomas. Assessment of CPD formation within strongly positioned nucleosomes using another published map of CPDs created by the HS-Damage-seq method [17] also produced an ~10 bp oscillation in CPDs across the nucleosome core particle (S3 Fig). However, the maximum of this periodicity was shifted ~5 bases resulting in CPDs occurring more frequently at inward facing dinucleotides in this data set and opposing the oscillation observed in melanoma mutations (S3J Fig). This shift is likely due to HS-Damage-seq under-representing CPDs in non-TT dipyrimidines [17]. TT dinucleotide sequences are over-represented at inward facing rotational settings in nucleosomes [35], indicating that the underlying sequence specificity of CPD formation is likely driving the oscillation in this data set. Supporting this, normalization of the HS-Damage-seq data set by dividing the in cell CPD formation data set by CPDs measured on UV-irradiated naked DNA shifts the oscillation towards favoring the outward facing dinucleotides (S3F Fig).

We then investigated the impact of lesion repair on the mutation distribution at nucleosome positions. We determined the positions of nucleotide excision repair products containing CPD lesions from previously published XR-seq sequencing reads generated from NHF1 cells isolated 1 hr, 4 hr, and 8 hr after treatment with 10J/m^2^ of UVC light [9]. Subsequently, we counted NER events at each nucleotide among strongly positioned nucleosomes and normalized this data for sequence effects by dividing the number of NER events by the number of CPDs formed in similar positions of naked genomic DNA treated with 20J/m^2^ of UVC light (determined by HS-Damage-Seq) [17] as well as by sequencing depth. HS-Damage-seq data was used to normalize the XR-seq values because XR-seq and HS-Damage-Seq follow a similar methodology and utilize an anti-CPD (Kamiya Biomedical, MC-062) antibody to enrich for lesion-containing DNA. Interestingly, NER activity at strongly positioned nucleosomes maintained an ~10 bp rotational pattern likely due to the increased amount of CPDs at outward facing dinucleotides resulting in higher amounts of repair at these sites. Despite the 10 bp oscillation, the most prominent period by Lomb-Scargle analysis occurs at ~112 bp (Fig 3E). This periodicity is almost the length of the nucleosome, suggesting that it may be caused by the translational position of the nucleosome inhibiting NER near the dyad. Supporting this, extending our analysis 500 bp in either direction beyond a central nucleosome dyad revealed an apparent ~150 bp oscillation consistent with the presence of neighboring nucleosomes (Fig 3F). Additionally, the repair events occurred with a positive translational curvature across the nucleosome, contrasting both CPD lesion formation and mutagenesis. Both the 10 bp oscillation and translational curvature occurred regardless of repair time point accessed (S4 Fig). These results indicate that the primary effect of nucleosome structure on NER efficiency is an inhibition of repair for events towards the nucleosome dyad position with greater accessibility to lesions occurring in DNA at the edges of the nucleosome core particle. Interestingly, while NER activity clearly oscillated with a 10 bp periodicity, the observed repair maxima and minima occur at positions in the nucleosome corresponding to the same maxima and minima sites as CPD formation and mutagenesis. This suggests that the periodicity is likely the result of changes in the frequency of lesion formation, which, in turn, influences the amount of repair activity at each nucleotide. Based on these results, we propose that the patterns of mutation across nucleosomes are established by two major processes: differential CPD formation, resulting in a 10 bp oscillation of mutation favoring outward-facing, more flexible dinucleotides, and decreased repair efficiency towards the center of the nucleosome core particle, which increases the density of mutations near the dyad.

### Chromatin state and histone modifications predict mutation density differences across nucleosomes

**Fig 4:**
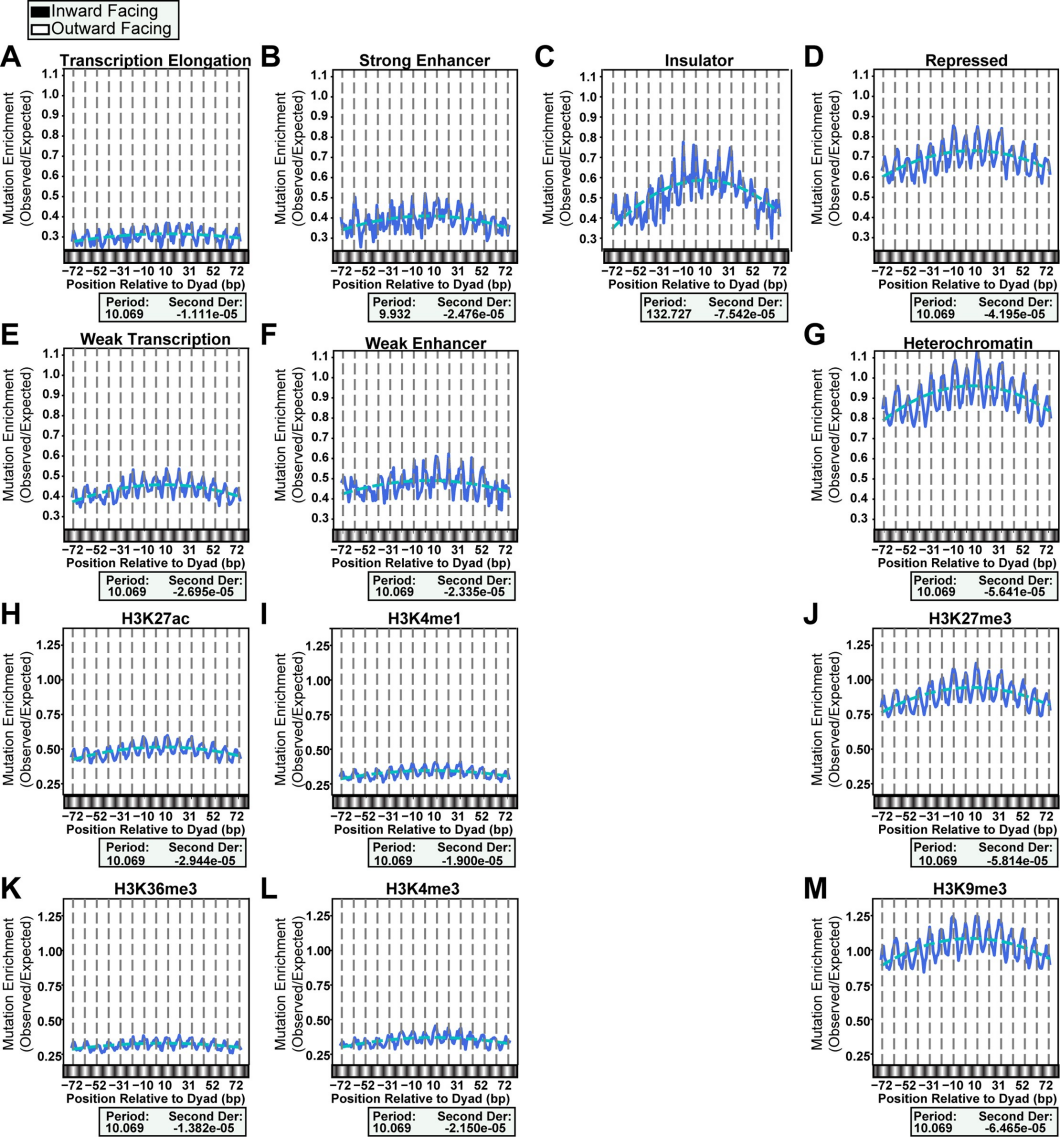
Mutations at nucleosomes parsed by chromatin state and histone modification. Mutations enrichments (solid blue lines) at nucleosomes across multiple chromatin states (*A-G*) and with different pre-existing histone modifications (*H-M*) show the same ~10 bp periodicity. Overall mutation enrichment varied widely across all chromatin states (p-value = 0.0014 by Kruskal-Wallis) and all histone modification-sorted nucleosomes (p-value = 2.55x10-6 by Kruskal-Wallis) with transcription elongation (*A*) and heterochromatin (*G*) and H3K36me3 (*K*) and H3K27me3 (*J*) being some the most different pairs (p-values = 0.0357 and 0.002, respectively by Dunn’s Test).

Since previous studies have shown globally that chromatin compaction correlates with mutation density, we sought to further classify the nucleosomes to see if their chromatin state altered the prominence of mutation periodicity and/or translational curvature. We analyzed mutation densities across nucleosomes parsed among chromatin states determined by the chromHMM software [29]. Only 7 of the 15 states contained an average of at least 100 mutations at each bp position across their respective composite strongly positioned nucleosome core particle, which we chose as a threshold to ensure sufficient statistical power to observe any mutation patterns. All of these remaining states displayed a mutational periodicity of ~ 10 bp across nucleosomes, associated with peaks in mutation density at outward facing dinucleotides (Fig 4A–4G). Apparent differences in the amplitude of the 10 bp oscillation between actively transcribed chromatin states and heterochromatin result from lower mutation numbers occurring in transcribed nucleosomes compared to heterochromatic nucleosomes and are not indicative of a greater difference in susceptibility of inward and outward facing dinucleotides to UV-induced damage and mutation in heterochromatic nucleosomes. Supporting this, when adjusted for equal sequencing depth among differentially modified nucleosomes, analysis of mCPD enrichment across strongly positioned nucleosomes with histone modifications indicative of active transcription (H3K27ac, H3K4me1, H3K36me3, and H3K4me3) or heterochromatin histone marks (H3K27me3 and H3K9me3) produced oscillations of similar amplitude (S5 Fig). These results confirm previous biochemical data indicating that no difference exists in either the UV-damage periodicity patterns or UV absorption strength of DNA in different chromatin condensation states [36]. In addition to the strong ~10 bp oscillation, a peak in mutation density near the nucleosome center, reflected in the overall negative curvature, was also present in all chromatin states analyzed, however the slopes of curvature and overall mutation densities varied significantly among different states (p-value = 0.0014; performed by inverting the axes, binning data, and using non-parametric ANOVA [Kruskal-Wallis]).

The chromatin states displaying the highest pairwise divergence in nucleosome-associated mutation density were between transcription elongation regions and heterochromatic nucleosomes (p-value = 0.0357; performed by Dunn’s Multiple Comparison) (Fig 4A and 4G). Nucleosomes within transcription elongation regions exhibited significantly lower overall mutation density and weaker curvature compared to the heterochromatic nucleosomes, possibly due to more efficient NER in the transcription elongation regions (i.e. due to transcription coupled-NER). These two states are defined by specific histone modifications that may themselves alter the generation of mutation oscillation and curvature across nucleosomes, either by specifically recruiting repair factors or modulating transcription. To determine the impact of individual histone modifications associated with these chromatin states, we acquired ChIP-seq data from the Epigenomics Roadmap Project [37] for histone marks H3K27ac, H3K27me3, H3K4me1, H3K4me3, H3K36me3, and H3K9me3 and determined the locations of nucleosomes containing these modifications using MACS2 software [26]. Consistent with the results obtained from broad chromatin states, the mutation densities in post-translationally modified nucleosomes showed ~10 bp oscillations and negative curvature, but a variety of curvature slopes and overall mutation densities across histone modifications (Fig 4H–4M) (p-value = 2.55x10^-6^ by Kruskal-Wallis). A striking difference in mutation density occurred between H3K36me3 and H3K27me3 (p-value = 0.002 by Dunn’s Test) (Fig 4K and 4J), which are canonically associated with high and low transcription of genes, respectively.

### Mutational curvature across nucleosomes decreases with increased transcription

**Fig 5:**
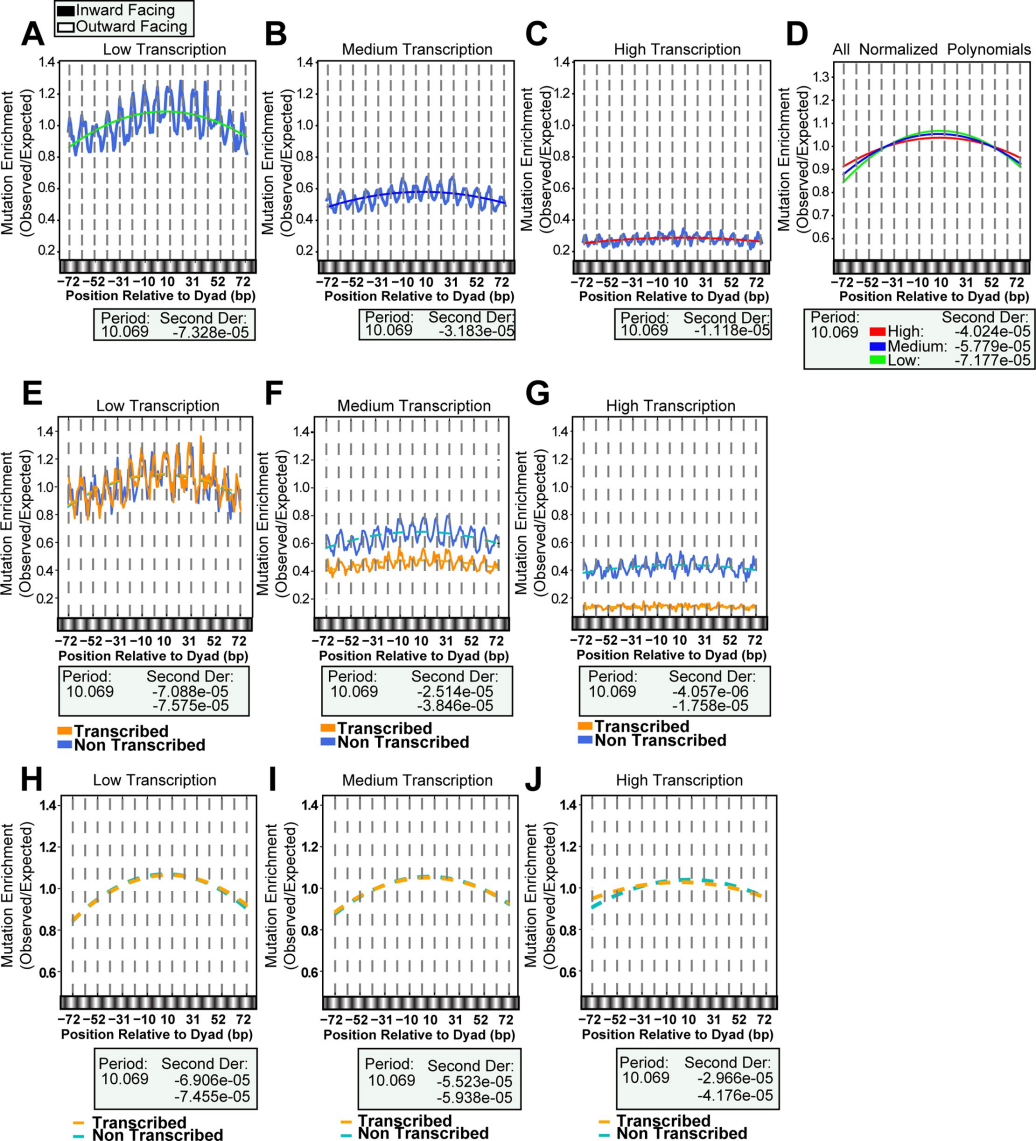
Mutations at nucleosomes parsed by transcription level. (*A-C*) Mutations counted across all transcription levels displayed significantly different enrichments (p-value = 3.90x10^-6^ by Kruskal-Wallis), with the most divergent being with High vs Low (p-value = 1.98x10^-6^ by Dunn’s Test). They all produced an ~10 bp periodicity, but appeared to have progressively more shallow curvature. (*D*) The mutations in each category were normalized by their respective average enrichment and best-fit second order polynomials were made for the data sets. Second derivatives were calculated to quantify the curvatures. Between High and Low the difference was almost 2-fold (respectively -7.177x10^-5^ and -4.024x10^-5^). (*E-G*) The same periodicity was also observed in both DNA strands despite different transcription levels and TC-NER activity occurring on the transcribed DNA strand. (*H-J*) While the mutation density changed between transcription levels, within each level the curvature appeared to be almost identical between strands.

Given that the most pronounced differences in mutation density based on chromatin states and histone modifications were also strong indicators of transcription, we hypothesized that transcription levels could be a major contributor to the curvature of mutation density across nucleosomes, especially due to the activity of TC-NER. We therefore repeated our mutation counting analysis with the nucleosomes sorted into high, medium, and low transcription level based upon their average RSEM RNA-seq level in 470 melanomas. We observed the same ~10 bp periodicity as in all previous analyses. However, as transcription level increased, mutation density decreased (p-value = 3.90x10^-6^ by Kruskal-Wallis), (Fig 5A–5C), as did the slope of the curvature in mutation density associated with the translational setting of the nucleosome. The apparent difference in curvature slope could result from lower numbers of mutations in highly transcribed regions reducing the potential change in slope of the best fit polynomial. We therefore normalized each density by their respective average mutation load and generated best-fit polynomials for the normalized densities. Quantification of these curvatures, by calculating the second derivative of each polynomial (Fig 5D), revealed a trend across transcriptional levels (second derivatives of -7.177x10^-5^, -5.779x10^-5^, and -4.024x10^-5^ for Low, Medium, and High transcription, respectively; p-value of 1.26x10^-4^ by Chi-Square between Low and High), showing an almost 2-fold reduction in the extent of curvature at high transcription levels compared to low transcription.

This difference in mutation curvature might result from differential repair due to changes in nucleosome occupancy as transcription increased. We therefore assessed the translational curvature of CPD lesion formation and repair at the transcription-parsed nucleosomes. Surprisingly, we observed no significant difference in the translational curvature of the normalized lesion or repair data between high, medium and low transcribed nucleosomes (S6 Fig), indicating the mutational process responsible for this difference in curvature may be independent of CPD lesion formation or repair. However, the transcribed strand (TS) of genes experiences transcription-coupled repair (TCR) meaning that analysis of NER capacity across nucleosomes could be confounded by differences in repair between DNA strands. Performing the same analysis of translational curvature of the melanoma mutations across nucleosomes, but differentiating between the TS and non-transcribed strand (NTS) of the genes, revealed an expected lower mutation density on the TS of nucleosomes as compared to the NTS (Fig 5E–5G). Additionally, both the TS and NTS showed decreased mutation density as transcription increased, which corroborated recent results indicating that transcription increased NER repair efficiency of both DNA strands in cutaneous squamous cell carcinoma [34]. However, the second derivatives of the normalized best fit polynomial describing the curvature of mutation density across the nucleosome indicated no difference existed between strands at any of the transcription levels (Fig 5H–5J). Thus, we are unable to detect a role for either CPD formation or CPD repair in generating the differences in mutational curvature across differentially transcribed nucleosomes.

## Discussion

**Fig 6:**
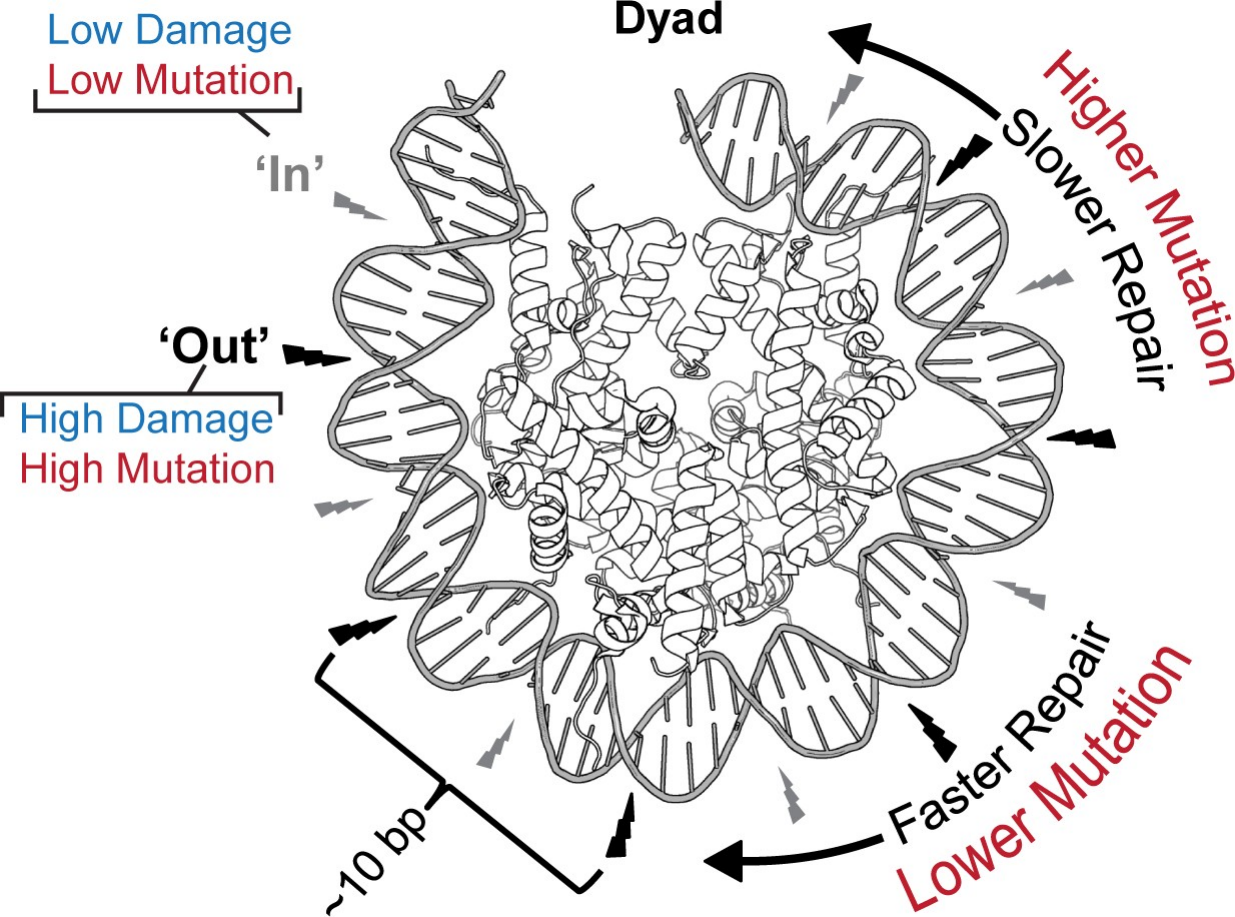
Mechanisms generating an extended UV-induced mutation signature within nucleosomes. Nucleosome structure increases CPD lesion formation at outward facing nucleosomes (black bolts) while decreasing NER activity near the dyad axis. Both processes combine to produce the 10bp oscillation of mutation across nucleosomes in melanoma as well as an overall curvature in mutation density across the translational setting of the nucleosome.

Recent whole genome studies have begun outlining the effects of chromatin states and TFs on where UV lesions form, NER efficiency, and how these effects contribute to mutational heterogeneity in human melamonas [9, 15–17]. Here, we use maps of CPD formation, NER activity, and UV-induced mutations from sequenced melanomas to elucidate the impact of the nucleosome on mutagenesis in cancer. Our focused analysis of mutations residing in strongly positioned nucleosomes revealed an epigenetic signature (beyond sequence context) of UV-induced mutations which fluctuates with an ~10 bp periodicity (Fig 6). This mutational pattern likely results from higher CPD formation at more flexible, outward facing dinucleotides as DNA is bent around the histone octamer [23]. Both CPDs measured by CPD-seq and NER activity measured by XR-seq also display an ~10 bp oscillation of similar magnitudes (Fig 3A and 3E), indicating that while CPDs preferentially form at outward facing dinucleotides, NER likely accesses lesions equally whether they occur at inward or outward facing positions. This agrees with our past report for CPD removal across nucleosomes in human cells [38]. While repair likely plays a lesser role in producing the observed periodicity, it appears to generate a curvature in mutation density across the length of the nucleosome. We believe this is the result of lesions near the edge of nucleosomes being more accessible to repair enzymes than those near the dyad. Nucleosome “breathing” (i.e. unwrapping-wrapping motion of DNA on the core histones), which has been shown both in models of nucleosome structural dynamics [39] and in *in vitro* accessibility assays [40], could provide NER enzymes greater accessibility to UV lesions in these locations. Alternatively, histone modifications or chromatin remodelers may play a role in making DNA at the edges of the nucleosome more accessible to the NER machinery.

Both chromatin states and histone modifications broadly correlate with differences in mutation density in a variety of cancers, including melanoma. However, these correlations appear to primarily result from effects derived by higher order structural organization of chromatin, as opposed to differences in the structure of individual nucleosomes. We saw expected differences in the overall number of mutations observed among nucleosomes within repressed and active chromatin states, as well as histone modifications, which are associated with repressed and active genes, respectively. Moreover, more mutations occurred on both the transcribed and non-transcribed stands of DNA as repression increased, which corroborated previous studies [34]. However, the 10 bp mutational periodicity associated with the rotational setting of nucleosomes was maintained regardless of the chromatin state, histone modification, or transcription level of the nucleosomes assessed. Thus, CPD formation appears to be unaltered by the specific modification or compaction state of the nucleosome and is only impacted by the fundamental wrapping of DNA around the histone octamer. In contrast, the degree of translational curvature of mutations differed among nucleosomes based upon chromatin state and histone modification. This effect could result from certain histone modifications facilitating the recruitment of NER proteins to the site of UV damage. H3K36 methylation has previously been shown to be involved in other DNA repair processes [41, 42]. Additionally, depletion of the acetyltransferase GCN5 in yeast reduces NER efficiency, suggesting that some interaction between the NER machinery and histone modification may exist [43–45].

Alternatively, H3K36 methylation and H3K9 trimethylation are markers of active and repressed transcription, respectively. The different transcription levels associated with these histone marks may facilitate repair near the dyad of nucleosomes by reducing histone occupancy in more highly transcribed regions. We did observe a decrease in mutational curvature across nucleosomes as their transcription level increased. Further examination of CPD-seq and XR-seq levels, however, indicated that no difference existed in the curvature of CPD formation or NER activity across the translational setting of the differentially transcribed nucleosomes. Thus, neither our analysis of lesion formation nor repair could account for the decrease in curvature of more highly transcribed nucleosomes. This effect therefore may originate from differences in the usage of trans-lesion synthesis polymerase η (which bypasses CPDs with high fidelity [46, 47]) or the rate of cytidine deamination [48] at CPDs in different chromosome contexts. The rotational setting of DNA in nucleosomes alters cytidine deamination rates of CPDs [49]. CPD-associated deamination may be similarly affected by the translational setting, especially considering that mutations caused by spontaneous cytidine deamination in yeast are elevated in linker regions between nucleosomes compared to nucleosome bound DNA [50]. Global approaches to mapping deamination rates in the future may allow for this supposition to be tested.

A complete understanding of the determinants of mutational heterogeneity in cancer will continue to provide important insights into the mechanistic processes that govern the efficiencies of lesion formation and DNA repair. We describe here an epigenetic regulation of lesion formation, repair, and ultimately mutagenesis by nucleosome structure, however, other chromosomal features additionally exacerbate mutational heterogeneity beyond that expected by sequence preferences for DNA damage. Transcription factor binding has clear impacts on lesion formation [16, 19] and strongly contributes to increasing mutation frequencies in melanomas [15, 18]. Likewise, the intrinsic curvature of DNA has also been recently reported to predict regional mutation differences in both yeast model systems and multiple human cancers including melanoma [51]. This impact of DNA curvature appears to relate to less curved sequences accumulating more DNA damage and mutagenesis. In contrast, the elevation of CPDs and UV-induced mutation at outward facing dinucleotides compared to inward facing dinucleotide clearly occurs in curved DNA induced by histone binding. These apparently contrasting results indicate that DNA damage occurring in different chromatin states (e.g. nucleosome bound, transcription factor bound, or unbound DNA) may influence which factors provide the dominant physical characteristic to influence the efficiency of mutagenesis. The integration of all these processes into different rates of mutation regionally, or even at a single nucleotide resolution, likely establishes the mutational heterogeneity observed in human cancers, which likewise impacts carcinogenesis by establishing high-risk sites within genomes that may harbor key cancer driver genes. As much of the differences in mutation rate are independent of selection by the tumor (as most mutations confer no advantage to the tumor), mutational heterogeneity also obscures our ability to differentiate selected driver events from mutagenic hotspots [2, 7]. Our recent determination that Ets family transcription factors greatly sensitize their binding sites to CPD formation, and ultimately mutation, highlights the potential difficulty in this determination [15]. Multiple sites, as exemplified by the Ets site in the *RPL13A* promoter, are highly recurrent in melanoma, but appear to be unlikely cancer drivers based on function of the gene regulated by the mutated promoter. The extended UV-induced lesion and mutation signature generated by nucleosome structure could produce similar effects, especially considering the large number of dinucleotides in the genome that reside at outward facing rotational settings in nucleosomes. The scope of these sensitive sites greatly expand the potential for strongly positioned nucleosomes to facilitate carcinogenesis by their shaping of the genomic mutational landscape.

## Materials and methods

All mutation, lesion, and repair data, as well as genomic coordinates for nucleosomes, chromatin states, histone modification peaks, and genes were analyzed using custom python3 scripts.

### Analyzing total melanoma and prostate mutations

Mutations from 184 melanoma samples were obtained from https://dcc.icgc.org/api/v1/download?fn=/release_20/Projects/MELA-AU/simple_somatic_mutation.open.MELA-AU.tsv.gz and from 216 prostate donors https://dcc.icgc.org/api/v1/download?fn=/release_25/Projects/PRAD-UK/simple_somatic_mutation.open.PRAD-UK.tsv.gz. Mutations occurring in multiple tumors from the same patient may have arisen before metastasis and were removed. Initial analysis of the impact on nucleosome position on mutation density (Fig 1) utilized all single nucleotide base substitutions.

### Determining nucleosome positions

Pre-computed nucleosome scores were acquired from [28]. A greedy algorithm was implemented in C++ to identify the central dyad positions of nucleosomes using the nucleosome scores. The algorithm employed a priority queue to select the next highest nucleosome score, after excluding all nucleosome scores for positions occurring within 117bp of called nucleosome dyads. Nucleosomes that overlapped with ENCODE blacklisted regions (Duke and DAC) were excluded. Strong nucleosomes had a score of 10 or greater and weak nucleosomes had scores between -5 and -40.

### Acral and cutaneous mutation parsing

Only single base pair mutations occurring in dipyrimidine contexts were used for analyses which were normalized by the expected number of mutations. The subtype of each tumor determined from Supplemental Table 1 in [3].

### Calculating expected mutations

The number of mutations in each possible trinucleotide context were counted and divided by the total number of mutations. Once these frequencies were obtained, the DNA sequences were acquired for each nucleosome and the calculated frequencies were applied to the trinucleotides in the DNA sequences to produce expected mutation counts. Expected mutations were recalculated for each subset analysis to correctly normalize the respective observed mutations. For analyses limited to mutations occurring in dipyrimidine contexts, expected values were likewise calculated only using trinucleotide contexts that contain dipyrimidines.

### Random sampling of cutaneous melanoma mutations

Subsets were generated using the “random” python3 module and randomly choosing ~1/100 of the cutaneous mutations. The ratio of T and C mutations was maintained by choosing proportional subsets from each mutation type. The Lomb-Scargle analysis was performed on each subset to identify the dominant periodicity. Periodicities greater than 100 bp were excluded to detect the presence of ~10 bp peaks.

### Determining the position of lesions in CPD-seq, XR-seq, and HS-Damage-seq data

For lesion formation and repair analyses, both the 5’ and 3’ positions of CPDs were used. CPD-seq data was acquired under accession number GSE103487 [15]. Raw sequencing reads for XR-seq data and HS-Damage-seq data were acquired from references [9] and [17] under accession numbers GSE76391 and GSE98025, respectively. The 1 hr, 4 hr, and 8 hr time points for repair of CPDs measured by XR-seq and the HS-Damage-seq of UV-exposed GM12878 naked DNA were used. These reads were mapped to the hg19 genome sequence using bowtie2 [52]. The position of lesions in XR-seq data was determined as in [15]. The HS-Damage-seq data was processed similarly, with the lesion position occurring 2 bp immediately 5’ of the read end as in [17]. The HS-Damage-seq CPD lesion positions were used for normalization of the XR-seq CPD lesion positions.

### Parsing nucleosomes by chromatin state, histone modification, and transcription level

Nucleosomes were sub-categorized by cross-referencing their positions with the genomic locations of different chromatin states, histone modifications, and transcription level. Chromatin states were acquired for the Nhlf cell line from [29]. Two “repetitive” states had low nucleosome counts (~less than 100 per state) and another 6 chromatin states had low mutation numbers (~less than 100 mutation per bp) and were thus removed from analysis. Location of histone modifications was determined from ChIP-seq data acquired from the Epigenomics Roadmap [37] for H3K27ac, H3K27me3, H3K4me1, H3K4me3, H3K36me3, and H3K9me3 (accession numbers GSM1127073, GSM958150, GSM958152, GSM958151, GSM958160, GSM958165, respectively). The MACS2 software package [26] was used to call peaks from the ChIP-seq data using standard parameters, with the additional stipulations of calling broad peaks with a p-value less than 0.01. The median expression level per gene for 470 human melanomas [30] was calculated from RSEM mRNA-seq data (http://gdac.broadinstitute.org/runs/stddata__2016_01_28/data/SKCM/20160128/gdac.broadinstitute.org_SKCM.Merge_rnaseqv2__illuminahiseq_rnaseqv2__unc_edu__Level_3__RSEM_genes_normalized__data.Level_3.2016012800.0.0.tar.gz). The CCDS gene positions (www.ncbi.nlm.nih.gov/projects/CCDS/CcdsBrowse.cgi) for the corresponding mRNAs were sorted by expression levels, and divided them into 4 quartiles: Low, Medium (the middle 2 quartiles), and High transcription.

### Statistical analyses and graphical representation

Statistical analyses were performed using python3, either with premade subroutines from python modules or personally designed analyses. The Lomb-Scargle analysis was conducted using the astropy module with default parameters. Second order polynomial (best-fit) functions were generated using a Least Squares method from the numpy module. Non-parametric ANOVA (Kruskal-Wallis) was performed using a subroutine modified from (https://gist.github.com/alimuldal/fbb19b73fa25423f02e8), as well as post-hoc Dunn’s test. Additionally, to generate distributions from the mutation data for the Kruskal-Wallis analysis, the axes of the data were inverted, where the enrichment values became positions along a continuous range and the bp positions became counts, tallied along the continuous range. The ends of the range were determined by identifying the maximum and minimum values of the combined data and rounding the enrichment (usually a decimal value) to the nearest integer. When plotted as a histogram the data sets showed features similar to normal distributions, and thus Kruskal-Wallis could be used to determine if their means were statistically different from one another. Chi-square was performed on the transcript-sorted nucleosomes by binning the observed mutations along the DNA sequence into ~10 bp bins (to remove the oscillatory effect; 16 bins total), and then performing the analysis between all pairwise combinations. Numerical values underlying graphs in the manuscript are provided in S1 Data.

## Supporting information

S1 Fig Strongly positioned nucleosomes are enriched in genomic locations with lower mutation densities. (*A*) The density of UV-induced melanoma mutations per nucleotide within strongly positioned nucleosomes (0.0058 mutations per nucleotide) and elsewhere in the genome (0.0071 mutations per nucleotide). Strongly positioned nucleosomes have reduced mutation density. (*B*) The expected number of mutations at each nucleotide across the 147 bp nucleosome core particle was calculated only using mutations occurring in strongly positioned nucleosomes (as opposed to all mutations across the genome as done in Fig 1) and used to normalize the observed mutations in dipyrimidine sequences in strongly positioned nucleosomes. Limiting the analysis to the subset of mutations occurring in strongly positioned nucleosomes results in enrichment values near 1, indicating expected and observed mutation counts are very similar.
(PDF)

S2 Fig Dominant periodicities for 1000 subsets of cutaneous mutations. To account for the 100-fold difference in mutations between acral and cutaneous subtypes, subsets were taken of the cutaneous mutations with ~100-fold fewer mutations. The mutations were then counted at strongly positioned nucleosomes, normalized to expected mutations, and were analyzed with Lomb-Scargle to determine periodicity. The occurrence of each periodicity was counted and revealed that 99.3% of the periodicities maintained a prominent ~10 bp.
(PDF)

S3 Fig Comparison of CPD oscillation across nucleosomes as measured by CPD-seq and HS-Damage-seq. (*A* and *B*) raw counts and enrichment measurements of total CPDs or (*C* and *D*) mCPDs by CPD-seq. (*E-H*) Similar analysis as A-D except measured by HS-Damage-seq. Overlay of raw counts for (*J*) all CPDs measured by HS-Damage-seq (blue line) and melanoma mutations (red line) or (*K*) mCPDs by CPD-seq (black line) and melanoma mutations (red line).
(PDF)

S4 Fig Repaired CPD lesions counted at nucleosome positions. The (*A*) 1 hr, (*B*) 4 hr, and (*C*) 8 hr in cells CPD repaired lesion counts normalized to *in vitro* CPD lesion counts (XR-seq by HS-Damage-seq; also normalized for read counts) at nucleosome positions appeared to produce identical patterns.
(PDF)

S5 Fig CPD formation measured across nucleosomes with different pre-existing histone modifications. Normalized CPD formation measured by CPD-seq was determined at each base pair across nucleosomes marked with pre-existing (*A*) H3K27ac, (*B*) H3K4me1, (*C*) H3K27me3, (*D*) H3K36me3, (*E*) H3K4me3, or (*F*) H3K9me3. CPDs (solid blue line) oscillate with similar periodicity and amplitude regardless of modification.
(PDF)

S6 Fig CPD lesion and repaired CPD lesion counts across transcription level-sorted nucleosomes. Strongly positioned nucleosomes were parsed by transcription level. (*A*) all CPD lesions or (*B*) mCPDs from cells irradiated with UV light (measured by CPD-seq) were counted and normalized by similarly acquired CPD lesions formed in UV-irradiated naked DNA. CPD repair events (measured by XR-seq) occurring at (*C*) 1hr, (*D*) 4hr, or (*E*) 8hr post UV-irradiation were counted and normalized by *in vitro* CPD lesions (measured by HS-Damage-seq). The data was normalized by their respective enrichments and second order best-fit polynomials were calculated for each transcription level for lesion formation and repair events. Second derivatives were also calculated to quantify the curvature of each best-fit polynomial. There appeared to be no significant difference across transcription levels for CPD lesion formation or CPD repair.
(PDF)

S1 Data Numerical values underlying graphs. Values used to generate graphs are provided in separate spreadsheets based on their corresponding figures.
(XLSX)
