# Supplementary figures and images for: Nucleosome positions establish an extended mutation signature in melanoma
Source: PLoS Genet. 2018 Nov 28;14(11):e1007823. doi: 10.1371/journal.pgen.1007823 (PMC6287878; doi:10.1371/journal.pgen.1007823)

Figure S1

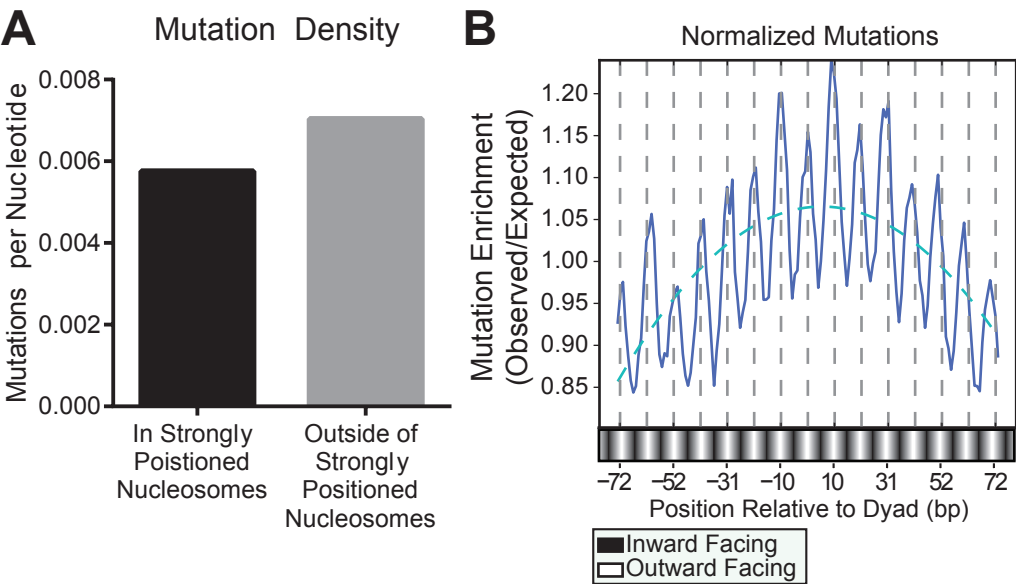

Supplement: S1 Fig — (A) The density of UV-induced melanoma mutations per nucleotide within strongly positioned nucleosomes (0.0058 mutations per nucleotide) and elsewhere in the genome (0.0071 mutations per nucleotide). Strongly positioned nucleosomes have reduced mutation density. (B) The expected number of mutations at each nucleotide across the 147 bp nucleosome core particle was calculated only using mutations occurring in strongly positioned nucleosomes (as opposed to all mutations across the genome as done in Fig 1) and used to normalize the observed mutations in dipyrimidine sequences in strongly positioned nucleosomes. Limiting the analysis to the subset of mutations occurring in strongly positioned nucleosomes results in enrichment values near 1, indicating expected and observed mutation counts are very similar. (PDF) [file pgen.1007823.s001.pdf]

Figure S2

1000 Subset  
Periodicities (bp)

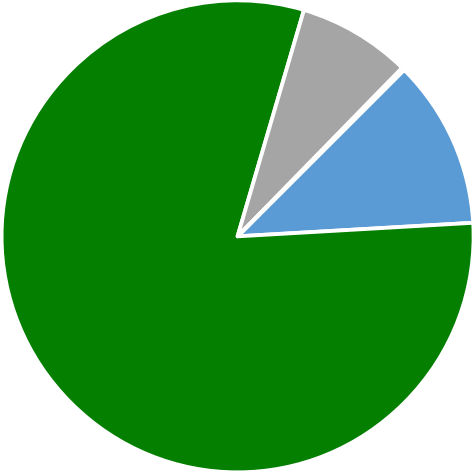

|                                                                                   | <u>Dominant<br/>Period</u> | <u>Times<br/>Observed</u> |
|-----------------------------------------------------------------------------------|----------------------------|---------------------------|
| 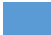 | 9.932                      | 115                       |
| 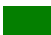 | 10.069                     | 799                       |
| 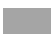 | 10.210                     | 77                        |
| 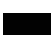 | 10.354                     | 1                         |
| 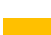 | 97.333                     | 8                         |

Supplement: S2 Fig — To account for the 100-fold difference in mutations between acral and cutaneous subtypes, subsets were taken of the cutaneous mutations with ~100-fold fewer mutations. The mutations were then counted at strongly positioned nucleosomes, normalized to expected mutations, and were analyzed with Lomb-Scargle to determine periodicity. The occurrence of each periodicity was counted and revealed that 99.3% of the periodicities maintained a prominent ~10 bp. (PDF) [file pgen.1007823.s002.pdf]

Figure S4

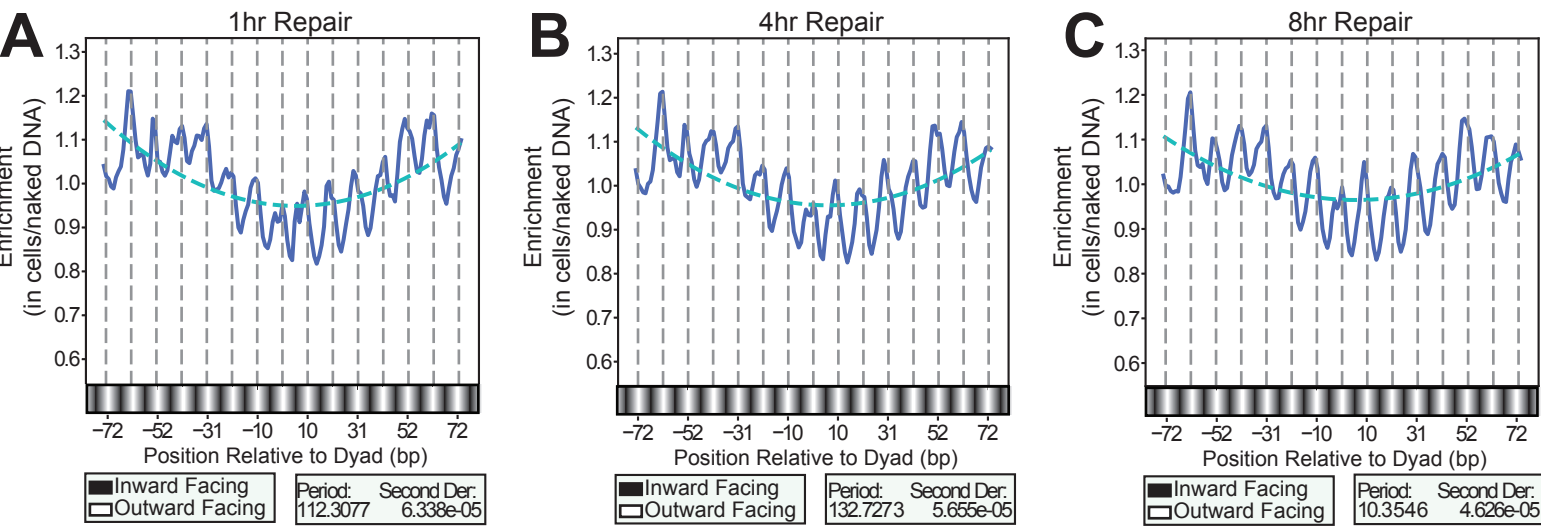

Supplement: S4 Fig — The (A) 1 hr, (B) 4 hr, and (C) 8 hr in cells CPD repaired lesion counts normalized to in vitro CPD lesion counts (XR-seq by HS-Damage-seq; also normalized for read counts) at nucleosome positions appeared to produce identical patterns. (PDF) [file pgen.1007823.s004.pdf]

Figure S5

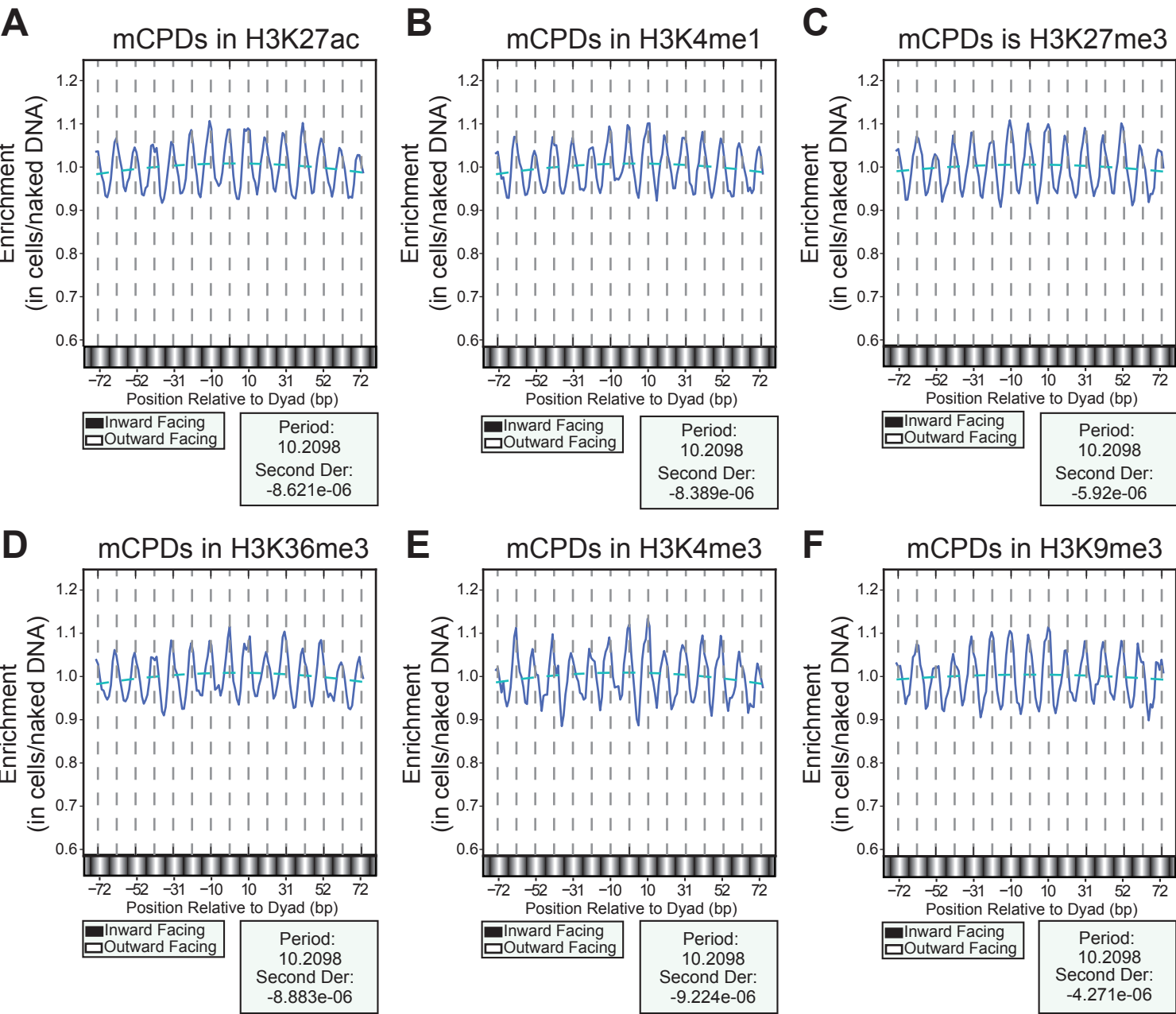

Supplement: S5 Fig — Normalized CPD formation measured by CPD-seq was determined at each base pair across nucleosomes marked with pre-existing (A) H3K27ac, (B) H3K4me1, (C) H3K27me3, (D) H3K36me3, (E) H3K4me3, or (F) H3K9me3. CPDs (solid blue line) oscillate with similar periodicity and amplitude regardless of modification. (PDF) [file pgen.1007823.s005.pdf]

Figure S6

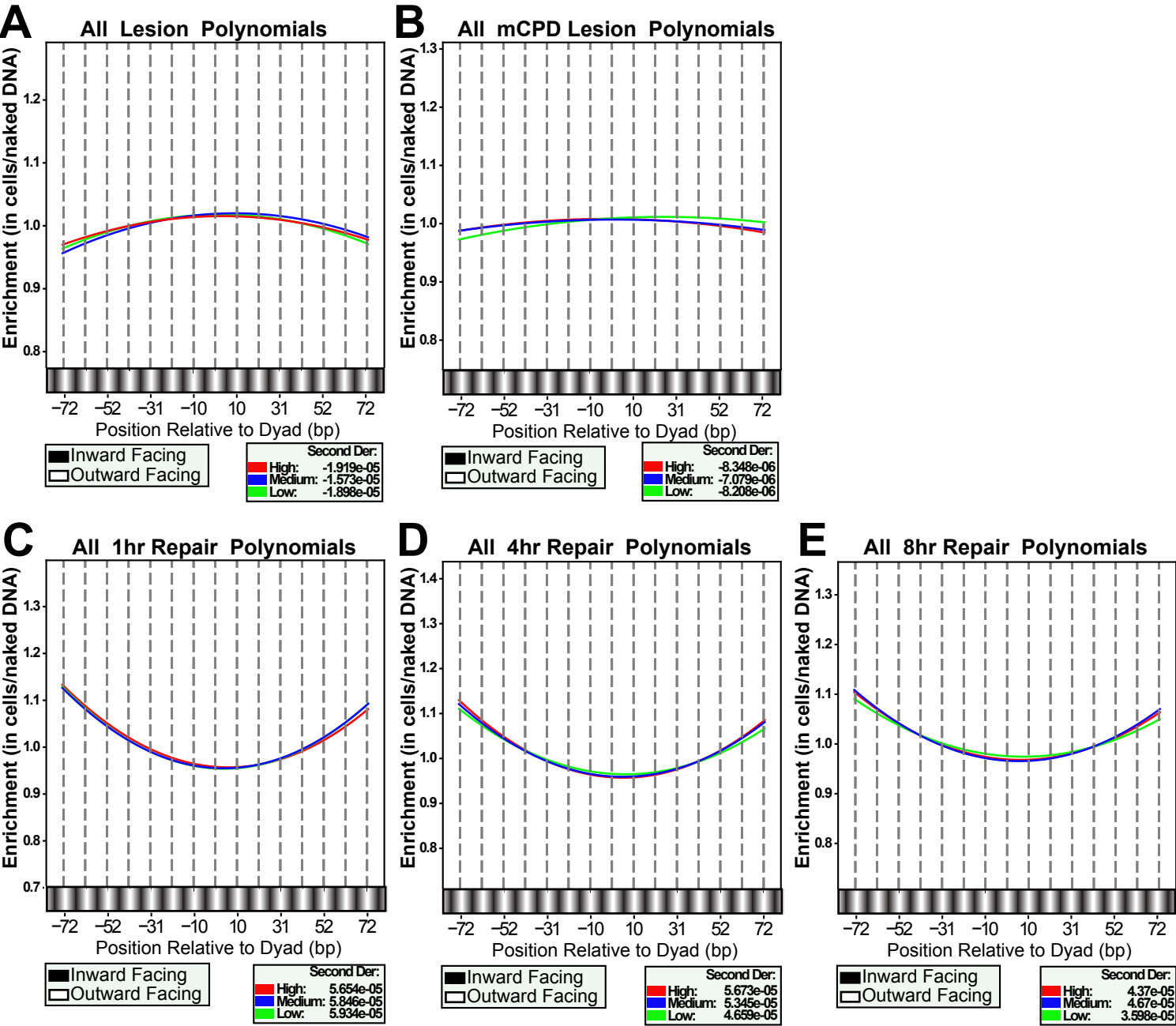

Supplement: S6 Fig — Strongly positioned nucleosomes were parsed by transcription level. (A) all CPD lesions or (B) mCPDs from cells irradiated with UV light (measured by CPD-seq) were counted and normalized by similarly acquired CPD lesions formed in UV-irradiated naked DNA. CPD repair events (measured by XR-seq) occurring at (C) 1hr, (D) 4hr, or (E) 8hr post UV-irradiation were counted and normalized by in vitro CPD lesions (measured by HS-Damage-seq). The data was normalized by their respective enrichments and second order best-fit polynomials were calculated for each transcription level for lesion formation and repair events. Second derivatives were also calculated to quantify the curvature of each best-fit polynomial. There appeared to be no significant difference across transcription levels for CPD lesion formation or CPD repair. (PDF) [file pgen.1007823.s006.pdf]
