# Supplementary material for: Nucleosome positions establish an extended mutation signature in melanoma
Source: PLoS Genet. 2018 Nov 28;14(11):e1007823. doi: 10.1371/journal.pgen.1007823 (PMC6287878; doi:10.1371/journal.pgen.1007823)
Supplement: S3 Fig — (A and B) raw counts and enrichment measurements of total CPDs or (C and D) mCPDs by CPD-seq. (E-H) Similar analysis as A-D except measured by HS-Damage-seq. Overlay of raw counts for (J) all CPDs measured by HS-Damage-seq (blue line) and melanoma mutations (red line) or (K) mCPDs by CPD-seq (black line) and melanoma mutations (red line). (PDF) [file pgen.1007823.s003.pdf]

Figure S3

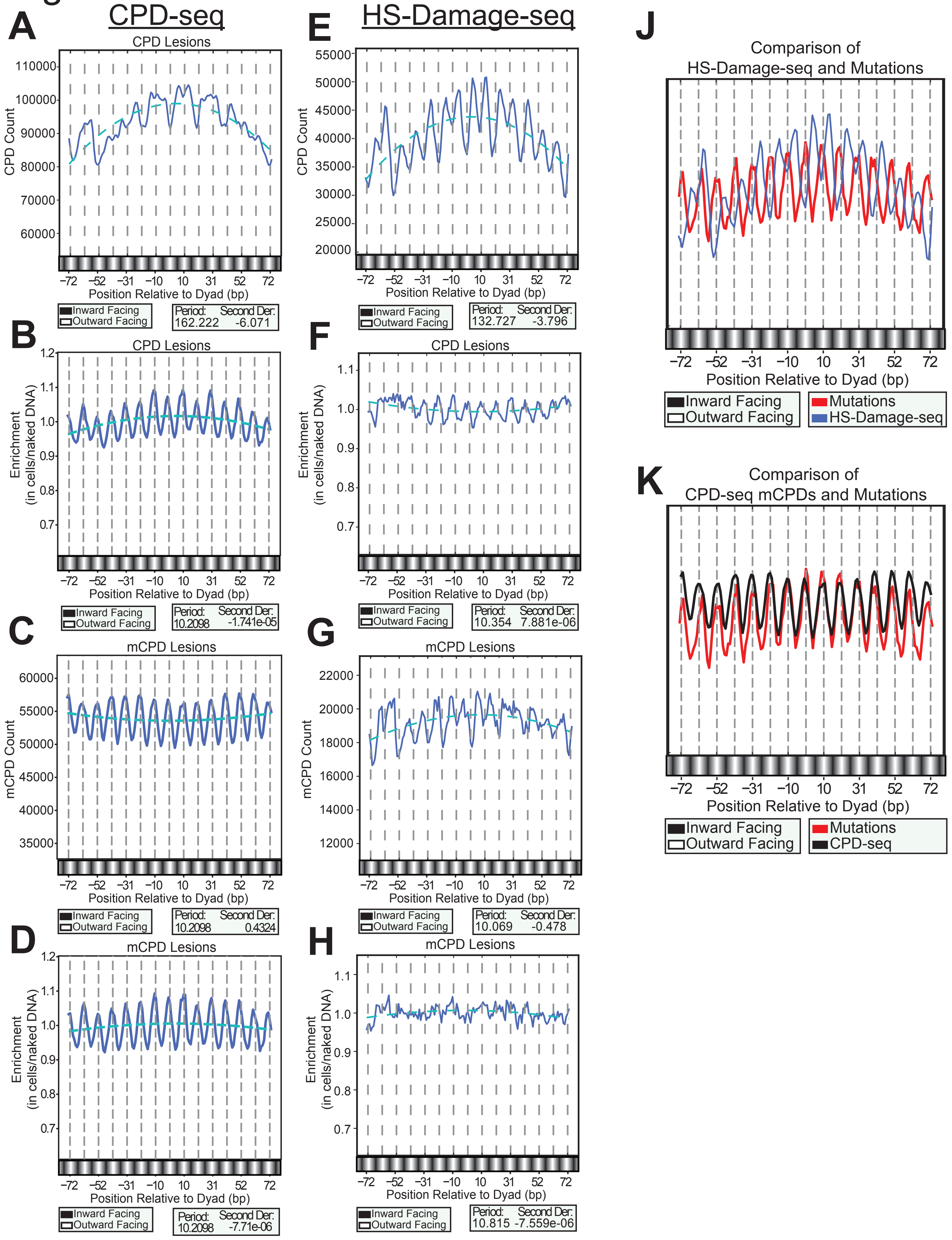
A
CPD-seq
CPD Lesions
CPD Count
Position Relative to Dyad (bp)
Inward Facing
Outward Facing
Period: 162.222 Second Der: -6.071
B
CPD Lesions
Enrichment (in cells/naked DNA)
Period: 10.2098 Second Der: -1.741e-05
C
mCPD Lesions
mCPD Count
Period: 10.2098 Second Der: 0.4324
D
mCPD Lesions
Period: 10.2098 Second Der: -7.71e-06
E
HS-Damage-seq
Period: 132.727 Second Der: -3.796
F
Period: 10.354 Second Der: 7.881e-06
G
Period: 10.069 Second Der: -0.478
H
Period: 10.815 Second Der: -7.559e-06
J
Comparison of HS-Damage-seq and Mutations
Mutations
HS-Damage-seq
K
Comparison of CPD-seq mCPDs and Mutations
Mutations
CPD-seq
